# Trafficking through COPII Stabilises Cell Polarity and Drives Secretion during *Drosophila* Epidermal Differentiation

**DOI:** 10.1371/journal.pone.0010802

**Published:** 2010-05-24

**Authors:** Michaela Norum, Erika Tång, Tina Chavoshi, Heinz Schwarz, Dirk Linke, Anne Uv, Bernard Moussian

**Affiliations:** 1 Interfaculty Institute for Cell Biology, University of Tübingen, Tübingen, Germany; 2 Institute of Biomedicine, Göteborg University, Göteborg, Sweden; 3 Max-Planck Institute for Developmental Biology, Tübingen, Germany; Institut Pasteur, France

## Abstract

**Background:**

The differentiation of an extracellular matrix (ECM) at the apical side of epithelial cells implies massive polarised secretion and membrane trafficking. An epithelial cell is hence engaged in coordinating secretion and cell polarity for a correct and efficient ECM formation.

**Principal Findings:**

We are studying the molecular mechanisms that *Drosophila* tracheal and epidermal cells deploy to form their specific apical ECM during differentiation. In this work we demonstrate that the two genetically identified factors *haunted* and *ghost* are essential for polarity maintenance, membrane topology as well as for secretion of the tracheal luminal matrix and the cuticle. We show that they code for the *Drosophila* COPII vesicle-coating components Sec23 and Sec24, respectively, that organise vesicle transport from the ER to the Golgi apparatus.

**Conclusion:**

Taken together, epithelial differentiation during *Drosophila* embryogenesis is a concerted action of ECM formation, plasma membrane remodelling and maintenance of cell polarity that all three rely mainly, if not absolutely, on the canonical secretory pathway from the ER over the Golgi apparatus to the plasma membrane. Our results indicate that COPII vesicles constitute a central hub for these processes.

## Introduction

Epithelia produce apical extracellular matrices (aECM) that are essential for their function as barriers. For this purpose, epithelial aECMs often adopt a tissue-specific and elaborate architecture. A central element of aECM formation is the apical plasma membrane that serves as an interface of aECM material delivery and as a platform for aECM organisation. Hence, along with deposition of aECM components into the extracellular space, the apical plasma membrane has to be equipped with factors that mediate its function during aECM differentiation. Both processes conceivably require concerted and polarised secretion and membrane trafficking.

Generally, secretion and membrane trafficking engage the basic secretory route running from the ER via coatamer protein complex II (COPII) coated vesicles to the Golgi apparatus, and from the Golgi apparatus via adaptor protein (AP)-clathrin-coated vesicles to the plasma membrane. This anterograde transport is usually counterbalanced by the retrograde transport of membranes from the plasma membrane to endosomes and the Golgi apparatus via AP-clathrin-coated vesicles, and from the Golgi apparatus back to the ER via COPI-coated vesicles. Selective docking of vesicles with their target membranes and their subsequent fusion both employ the activity of membrane-specific SNARE proteins [1]. These generic mechanisms are probably not sufficient to explain directionality of secretion. In polarised cells, directionality of vesicle transport depends on the cytoskeleton that is organised by subunits of protein complexes arranged along the apical and lateral plasma membrane [2], [3]. The evolutionary conserved transmembrane protein Crumbs (Crb) has an influence on the organisation of the actin cytoskeleton at the apical portion of the cell through the interaction with the actin-binding factor β-heavy spectrin [4], [5]. The stability of microtubules is regulated by the atypical protein kinase C (aPKC), which additionally manipulates the function of Crb [6], [7]. The cytoskeleton in turn stabilises the protein complex that constitutes the adherens junctions, which being basal to the subapical Crb-complex contribute to the tautness of epithelia. Finally, positioning and function of the Crb-complex is also regulated by the exocyst complex subunit Exo84 and by membrane recycling driven by the endosomal small GTPase Rab11 [8], [9].

While both the mechanisms of polarised secretion and the histology of various aECMs have been studied in detail, a link between polarised secretion in epithelia and aECM production is almost unexplored. An amenable tissue allowing detailed molecular and cellular analysis of aECM differentiation is the larval skin of the fruit fly *Drosophila melanogaster*. It consists of the epidermis and the apical cuticle that is an aECM deposited during embryogenesis. The *Drosophila* larval cuticle is a typical arthropod cuticle that adopts a stereotypic layered architecture composed of the polysaccharide chitin, lipids and proteins [10]. Several factors playing essential roles during *Drosophila* larval skin differentiation have been genetically identified and phenotypically characterised in the past few years. Most of these factors act within the apical plasma membrane. These are the Zona Pellucida (ZP) proteins Piopio (Pio) and Papillote (Pot) that mediate the contact between the aECM and the surface of the epidermal cells [11], and Retroactive (Rtv) and Knickkopf (Knk) that are required for the organisation of the chitin microfibril in the aECM, the molecular functions of which, however, are unknown [12], [13]. Mutations in the genes coding for these factors cause the detachment of the macroscopically normal-looking cuticle from the epidermis. A second group of mutations provokes a thin and pale cuticle suggesting a fundamental function of the affected genes in cuticle deposition. Some of these genes code for enzymes catalysing the synthesis of the steroid hormone ecdysone [14], while others encode factors involved in basic cellular processes related to secretion. One of these factors is Mummy (Mmy), the *Drosophila* UDP-GlcNAc pyrophosphorylase producing UDP-GlcNAc, which is an important component of N-glycans and the monomer of chitin [15], [16], [17]. Another genetically characterised factor that is crucial for secretion is the *Drosophila* Syntaxin1A (Syx1A), the SNARE of the apical plasma membrane [18]. Interestingly, mutations in *syx1A* abrogate the shape of the apical plasma membrane and the secretion of cuticle proteins, but not chitin synthesis that occurs at the plasma membrane suggesting that a second apical SNARE is needed for full aECM production.

Many of the same genes have been shown to be required for embryonic morphogenesis of the respiratory organ (trachea), a network of fine epithelial tubes that spans the organism. Newly formed tracheal tubes have a narrow lumen diameter that must expand several-fold before becoming functional. The widening of the tracheal lumen requires apical cell secretion [19], [20], and uniform diameter expansion depends on a transient chitinous luminal matrix [21], [22]. Both Knk and Rtv are needed for the organization of luminal chitin matrix, as later of the tracheal cuticle, and loss of *mmy* abolishes chitin production for either matrix [13], [15], [16].

For an advanced understanding of the molecular mechanisms governing aECM differentiation in polarised epithelial cells, identification and characterisation of additional factors is needed. Two genetically defined genes *ghost* (*gho*) and *haunted* (*hau*) are excellent candidates for this goal, as larvae suffering mutations in these genes display a phenotype similar to that of *syx1A* and *mmy* mutant larvae [23], [24]. In the present work, we show that *hau* and *gho* code for the *Drosophila* COPII components Sec24 and Sec23, respectively. We demonstrate that Hau and Gho function to maintain correct localisation of cell polarity markers in the tracheae and the epidermis while they support deposition of tracheal luminal and cuticle material. These findings underline that the secretory pathway is an important motor for the differentiation of the tracheae and the epidermis.

## Materials and Methods

### Fly work

Flies were kept in cages on apple juice agar plates at 25°C to collect. Embryos were staged according to the time of development at 25°C described in Hartenstein and Campos-Ortega [25]. Stocks used in this work are listed in table 1. Homozygous or transheterozygous mutant embryos or larvae were unambiguously and manually collected in a population of progeny segregating GFP-positive (Kr-Gal4 and UAS-GFP harbouring balancer [26]) and GFP-negative (mutant) embryos.

**Table 1:** Fly stocks used in this work.

genotype	origin
*Samarkand (wild-type)*	Bloomington
*hau^9G14^*	[23]
*hau^CK^*	[35]
*Df(3R)ED5187*	Bloomington
*gho^IB104^*	[24]
*gho^IP107^*	[24]
*Df(2L)BSC688*	Bloomington
*Df(2L)Exel7010*	Bloomington
*sar1^#28^*	[44]

Stocks of flies segregating the *hau^9G14^ a*nd both *gho* mutations were obtained from the Tübingen Stock Collection (http://www.eb.tuebingen.mpg.de/departments/3-genetics/drosophila/drosophila-stock-collection/drosophila-stock-collection). The *hau^CK^* segregating flies were generated by Kay Giesen in the laboratory of Christian Klämbt (Münster University). The *sar1* stock was a kind gift of Christos Samakovlis (Stockholm University). Flies that segregate deficiencies in the *hau* or *gho* chromosomic region were obtained from the Bloomington Stock Center (Indiana University, Bloomington, USA). All mutant stocks were kept over respective balancers carrying Kr-Gal4 and UAS-GFP [26].

### Microscopy

**Figure 1:**
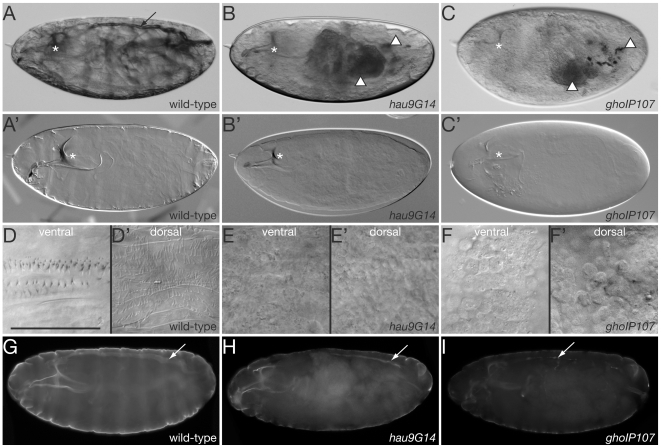
Larvae carrying mutations in *hau* and *gho* have a thin and pale cuticle. Wild-type larvae ready to hatch have a body cuticle that shrouds the inner organs (A). The head skeleton (*) and the air-filled dorsal trunk (arrow) are nevertheless discernable. By Nomarski microscopy, light refraction reveals the body cuticle of wild-type larvae within the egg case fixed in Hoyer's medium (A’). Due to a thin cuticle, the inner organs, such as the Malpighian tubules and the digestive system (arrowheads) of larvae mutant for *hau* and *gho* are well visible (B and C). Probably due to the failure to air-fill, their dorsal trunk is not identifiable at this magnification (compare to Figure 7). As seen in Hoyer's fixed *hau* and *gho* larvae, the mutant cuticle only weakly refracts light in Nomarski optics (B’ and C’). The head skeleton of these larvae seems to have a correct morphology but is less tanned. The ventral side of the wild-type larval body is decorated by belts of denticles, while more filigree hairs cover the dorsal side (D and D’). The ventral and dorsal sides of *hau* and *gho* mutant larvae, by contrast, are naked (E–F’). The surface of both mutant larvae is, however, not smooth but wrinkly, and in *gho* mutant larvae, epidermal cells at both sides appear to round up and leave the epithelium (see Figure 3). The epidermis, the dorsal trunk (arrow) and the head skeleton of wild-type stage 17 embryos are lined by chitin as detected with the FITC-conjugated chitin-binding probe (green, G). The chitin signal in the head skeleton and the body of *hau* and *gho* mutant stage 17 embryos is weaker than in wild-type embryos (H and I). Moreover, their dorsal trunk (arrow) is narrower than the wild-type one. (A–F’) Nomarski light microscopy of wild-type, *hau* and *gho* mutant larvae within the egg case. (G–I) Fluorescence microscopy of heat fixed stage 17 embryos. Scale bar in (D) is 50µm and applies to (D–F’).

For light and fluorescence microscopy, embryos were fixed chemically (in 3,7% formaldehyde) or physically (by boiling) according to standard protocols [27]. For immunohistochemical detection of antigens, the following primary antibodies were used in this study: the tracheal luminal specific mouse IgM monoclonal antibody 2A12 (1∶10, Developmental Studies Hybridoma Bank, DSHB), mouse IgG monoclonal anti-GM130 (1∶500, Abcam), mouse IgG monoclonal anti-KDEL (1∶400, Stressgen Bioreagents, Figure 9 or 1∶500, KR-10, Abcam, Figure 8), rabbit anti-Rab5 (1∶1000, [28]), rabbit anti-Rab11 (1∶8000, [28]), rabbit polyclonal anti-Verm (1∶300), and rabbit polyclonal anti-Knk (1∶1500, preabsorbed against wild-type embryos before use). A fluorescein-conjugated chitin-binding probe was used to detect chitin (CBP, 1∶500, New England Biolabs). For visualisation, secondary fluorescent antibodies from Molecular Probes (1∶500) were used: Alexa 488 goat anti-mouse IgM, Alexa 568 goat anti-mouse IgG, Alexa 568 goat anti-mouse IgG2a, Alexa 488 goat anti-rabbit IgG, Alexa 555 goat anti-rabbit IgG and Alexa 568 goat anti-mouse IgG1. A Nikon eclipse E1000 microscope was used for Nomarski and fluorescence imaging and Bio-Rad Radiance 2000 for confocal imaging.

Embryos and first instar larvae were prepared for electron microscopy following previously described protocols [29].

### Molecular biology

For identification of *hau* and *gho* (PCR & sequencing), molecular experiments were performed following standard protocols for molecular biology. For Western blot analysis, larvae were homogenized for protein isolation in PLC buffer containing protease inhibitors. Protein amounts were estimated by spectrometry at 280 nm [30]. Following SDS-polyacrylamide gel electrophoresis (SDS-PAGE, 7.5%), proteins were transferred to a nitrocellulose membrane (Whatman) by the semi-dry method. Proteins were detected using the Odyssey infrared dual-colour detection system (LI-COR® Biosciences). For immunodetection of Knk and Serp on Western blots, specific primary antibodies were used at the dilutions of 1∶1000 and 1∶300, respectively.

## Results

### Mutations in hau and gho affect cuticle differentiation

The wild-type first instar larval cuticle lines the body of the animal, structures its head, and stabilizes the air-filled trachea (Figure 1A,A’). Embryos mutant for either *haunted* (*hau*) or *ghost* (*gho*) produce only a discontinuous or thin larval cuticle and the tracheae do not become air-filled and are barely visible, whereas their head skeleton albeit less melanised, has a normal morphology (Figure 1B,B’ and C,C’). These animals die at the first instar larval stage within the egg case. Specialised cuticular structures such as the ventral denticles and the dorsal hairs are missing in *hau* and *gho* mutant larvae (Figure 1D–F). To test whether the epidermis and the trachea nevertheless produce chitin, we detected chitin with a FITC-coupled chitin-binding protein (CBP) in stage 17 wild-type and mutant embryos (Figure 1G–I). Mutations in either *hau* or *gho* cause reduced chitin in the epidermis and the trachea. Overall, the *gho* phenotype is stronger than the *hau* phenotype. Of note, larvae homozygous for any *hau* (*9G14* and *CK*) or *gho* (*IB104* and *IP107*) mutation or carrying the mutation in trans over each other or over respective deficiencies (see below) exhibit the same defects. We can therefore neglect the possibility that additional mutations on the chromosomes carrying *hau* or *gho* contribute to the strength of the phenotypes described. Taken together, *hau* and *gho* are required for correct deployment of cuticle melanisation and chitin deposition.

**Figure 2:**
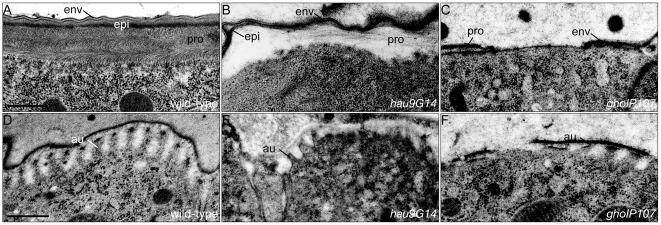
Hau and Gho are required for full cuticle differentiation. The wild-type larval cuticle is a stratified extracellular matrix (A). Based on the molecular composition, it is subdivided into three layers. The envelope (env) is the outermost layer, separated by the bipartite epicuticle (epi) from the innermost procuticle (pro). The cuticle of *hau* mutant larvae is disorganised (B). The electron-dense basal sublayer of the epicuticle often contacts the envelope and the chitin matrix has lost its tight packaging. In *gho* mutant larvae, the cuticle is fragmented and thin (C). Between late stage 16 and mid-stage 17 the apical plasma membrane of epidermal cells forms regular corrugations called apical undulae (au), at the tip of which chitin synthesis takes place, while secretion occurs at the valley between the corrugations (D). The epidermal cells of *hau* and *gho* mutant embryos fail to form repeated corrugations (E and F). (A–F) Electronmicrographs of ultrathinsections. Scale bar in (A) is 500nm and applies to (B) and (C). Scale bar in (D) is 500nm and applies to (E) and (F).

To understand the cellular defects caused by mutations in *hau* and *gho*, we examined the ultrastructure of epidermal cells in *hau* and *gho* mutant embryos and larvae. In the wild-type larva the cuticle consists of three biochemically distinct horizontal layers (Figure 2A). The outermost layer is the envelope that is composed of alternating electron-dense and electron-lucid films. The middle epicuticle is a bipartite proteinaceous layer with an upper electron-lucid and a lower electron-dense sublayer. An ordered chitin-protein matrix constitutes the innermost procuticle. The cuticle of *hau* mutant larvae has a reduced epicuticle, and the chitin-protein organisation is lost in the procuticle, while the envelope has a normal appearance (Figure 2B). The cuticle of *gho* mutant larvae is very thin and fragmented and lacks the epicuticle (Figure 2C).

Organisation of the procuticle is supposedly controlled by the apical plasma membrane that forms longitudinal corrugations called *apical undulae* during chitin synthesis that run perpendicular to the anterior-posterior axis of the developing embryo (Figure 2D) [29]. The epidermal cells of *hau* and *gho* mutant embryos usually do not establish apical undulae (Figure 2E,F). In rare cases, the apical plasma membrane of the epidermis of *hau* mutant embryos forms apical undulae close to the apical cell-cell junctions. In summary, *hau* and *gho* are needed for cuticle formation and shaping the epidermal apical plasma membrane.

### Hau and gho are needed for epidermal cell shape and polarity

**Figure 3:**
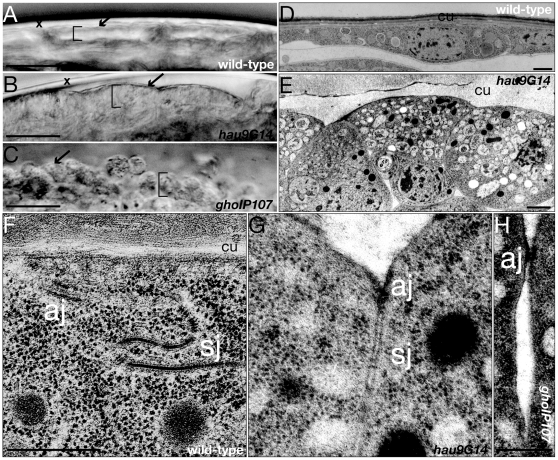
Hau and Gho are involved in shaping the larval epidermal cell. The cuticle (arrow) lines the apical side of the epidermis (bracket, A). The cuticle of *hau* and *gho* mutant larvae is thinner and discontinuous (arrows, B and C). Their epidermal cells (bracket) are cuboidal (B) or round and may lose contact to their neighbours (C, see also Figure 1F,F’). The wild-type larval epidermal cell is flat with large apical surface covered by the cuticle (cu, D). Epidermal cells of *hau* mutant larvae are cuboidal and their lateral membranes are straight (E). Epidermal cells of *gho* mutant larvae display the same phenotype (not shown). The wild-type epidermal cell contacts its neighbours with its meandering lateral membrane (F). Histologically, the two obvious contact features are the subapical adherens junctions (aj) and the lateral septate junctions (sj). The lateral membranes of epidermal cells of *hau* mutant larvae are straight and the junctions appear less prominent (G). Epidermal cells of *gho* mutant larvae show a similar phenotype (not shown). Occasionally and especially in the *gho* mutant larval epidermis, the cell-cell contacts are lost (H). (A–C) Light-microscopy of living wild-type, *hau* and *gho* mutant larvae within the egg case (x). (D–H) Electronmicrographs of ultrathinsections. Scale bar in (A–C) is 25µm. Scale bars in (D and E) are 1 µm. Scale bar in (F) is 500 nm and applies also to (G). Scale bar in (H) is 500 nm.

The epidermis of larvae mutant for *hau* and *gho* eventually disintegrates and single cells leave the tissue (Figure 3A–C). To have a more detailed understanding of this defect, we studied the morphology of the epidermal cells by electron microscopy. The wild-type larval epidermal cells are flat and contact their neighbours via a meandering lateral membrane that is mainly characterised by the apical adherens junctions and the basolateral septate junctions (Figure 3D,F). The *gho* or *hau* larval epidermal cells are cuboidal and their lateral membrane does not meander (Figure 3E,G,H). The adherens junctions of *hau* and *gho* mutant larval epidermal cells look loose and the basolateral septate junctions appear to be less complex. Especially in *gho* mutant larvae, the lateral cell-cell contacts are effaced (Figure 3H). At the onset of cuticle differentiation, at stage 15 cell shapes in *hau*, *gho* and wild-type embryos are indistinguishable (data not shown).

**Figure 4:**
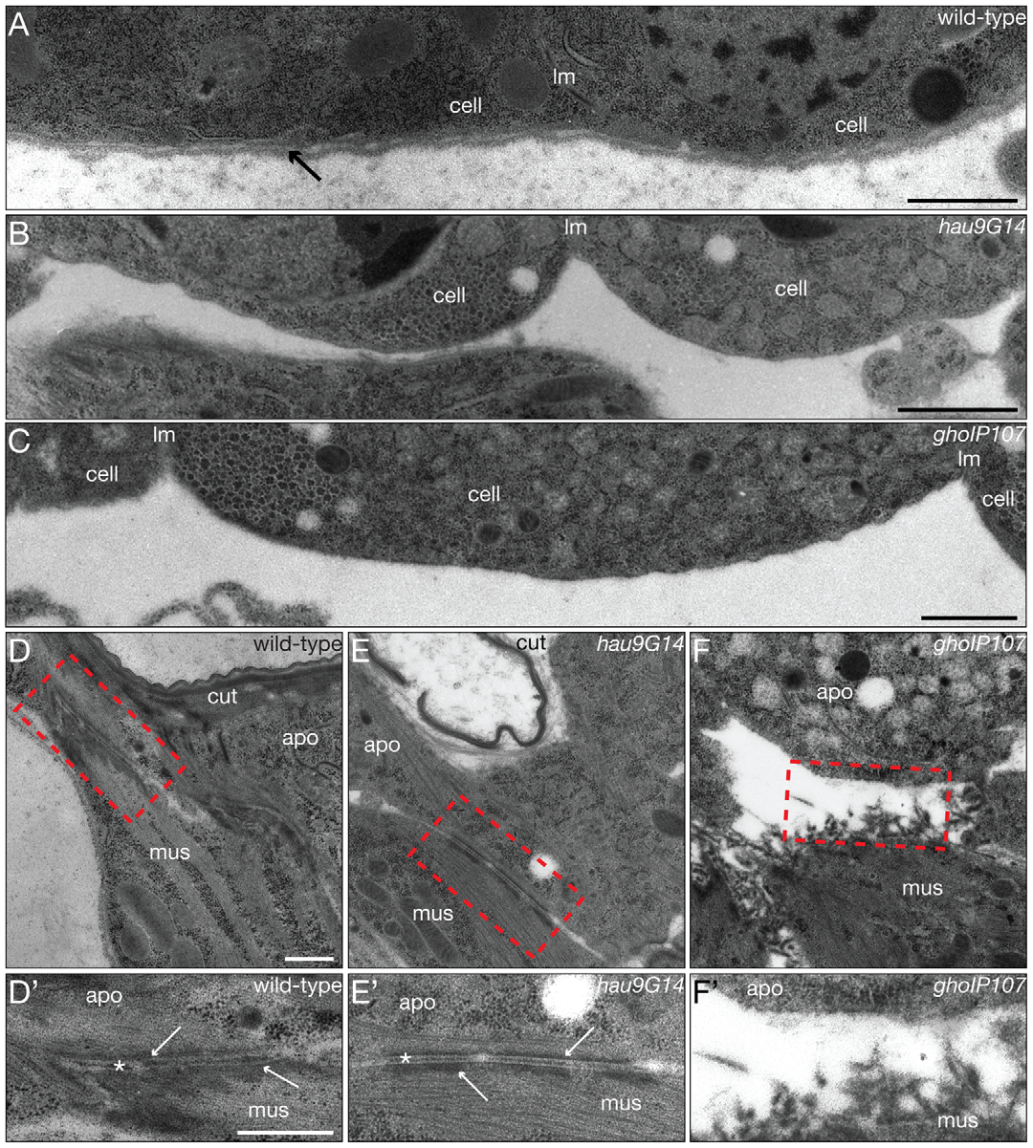
Hau and Gho conduct basement membrane production. The basal side of the wild-type larval epidermal cells (cell) separated by the lateral plasma membrane (lm) is underlain by the extracellular basement membrane (arrow, A). The basement membrane is missing in *hau* and *gho* mutant larval epidermal cells (B and C). The basal ECM (*) of wild-type larval apodemal cells (apo) is mediating the contact to muscles (mus) attached to the epidermis (D,D’). At the inner side of the apodemal cell and muscles electron-dense junctional material accumulates (arrows). The *hau* mutant larval apodemes have a normal-looking basal ECM, the muscular intracellular junctional material, however, is less abundant (E,E’). The *gho* mutant larval apodemal basal ECM is disrupted, and muscles detach from the epidermis (F,F’). No junctional material is detected at the inner side of the apodemal cell of these larvae. The red dashed rectangle in (D–F) is enlarged in (D’–F’). (A–F’) Electron micrographs of ultrathinsections. Scale bars in (A–C) are 1µm. Scale bar in (D) is 500nm and applies also to (E) and (F). Scale bar in (D’) is (500nm and applies also to (E’) and (F’).

As in many epithelia, the basal side of wild-type epidermal cells is covered by the basement membrane (Figure 4A). The basal side of *hau* and *gho* mutant epidermal cells is, by contrast, naked (Fig 4B,C). Defects at this side of *hau* and *gho* mutant epidermal cells have a more drastic effect in the muscle attachment sites, the apodemes (Figure 4D–F’). Muscles contact the jagged basal plasma membrane of apodemal cells by specialised junctions, the basal hemidesmosomes in wild-type larvae and a complex intracellular ECM (Figure 4D). In *hau* and *gho* mutant apodemes the basal plasma membrane is smooth (Figure 4E–F’), and the muscle often detaches from the epidermis (Figure 4F,F’).

**Figure 5:**
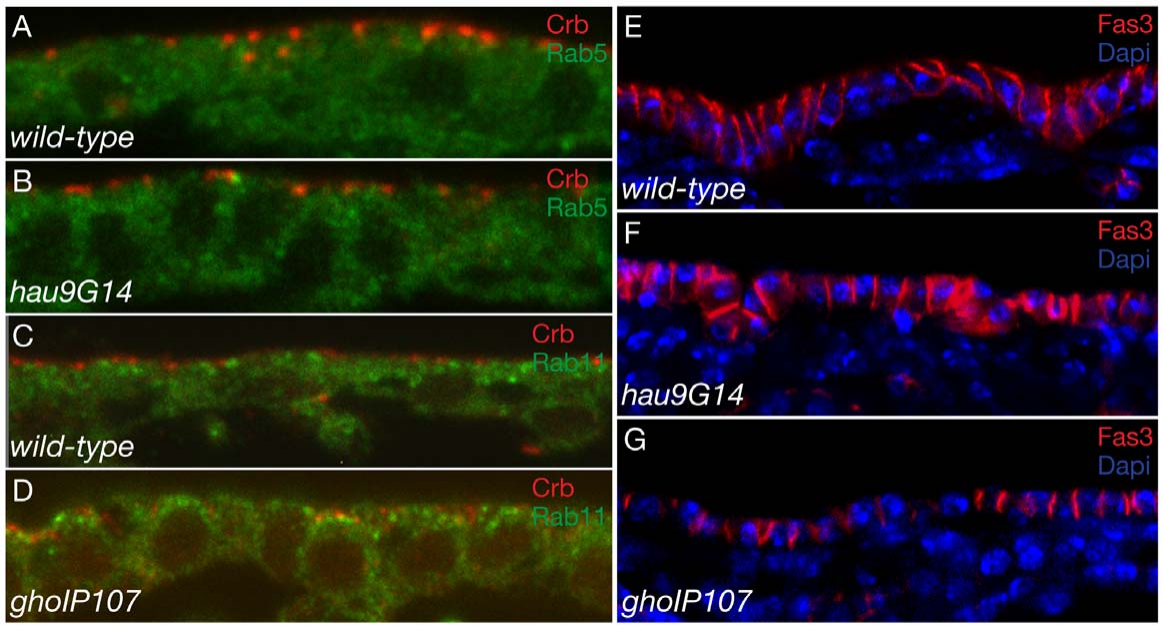
Hau and Gho stabilise epidermal cell polarity. The transmembrane protein Crb (red) localises to the apico-lateral region of wild-type stage 15 embryonic epidermal lateral plasma membrane (A). The small GTPase Rab5 (green) is distributed in the cytoplasm. Localisation of Crb and Rab5 is normal in *hau* mutant embryonic epidermal cells (B). At stage 16, Crb continues to localise to the apico-lateral region of the lateral plasma membrane (C). The small GTPase Rab11 (green) is distributed in the cytoplasm with a considerable accumulation at the apical portion of the cell. The Crb signal is detected also in the cytoplasm of *gho* mutant embryonic cells, while Rab11 distribution is normal (D). The lateral plasma membrane of wild-type embryos – here stage 16 - is marked by proteins like Fas3 (red) constituting the septate junctions (E). The Fas3 signal gradually decreases from the apical to the basal end of the lateral plasma membrane. The nuclei (blue) of these cells locate to the basal side of the columnar cell. In *hau* and *gho* embryonic mutant epidermal cells the Fas3 signal is homogeneously distributed along the lateral plasma membrane (F and G). The epidermal cells are cuboidal and the nuclei are lie in the middle of the cell. Images from confocal microscopy.

The ultrastructural studies indicate that cell polarity is compromised in *hau* and *gho* mutant larvae. To verify whether cell polarity may already be perturbed during the time of massive cuticle production, we performed immunohistochemical experiments in stage 15–17 embryos using antibodies against factors that mark different domains of the lateral plasma membrane. Crumbs (Crb) is a transmembrane protein and localises to a lateral position of the apical plasma membrane of epidermal cells in stage 15 and 16 wild-type embryos (Figure 5A,C) [4]. Crb localisation is normal in stage 15 *hau* mutant embryos (Figure 5B). In stage 16 *gho* mutant embryos, in addition to a variable but occasionally normal localisation at the apico-lateral membrane, the Crb signal accumulates within the cell (Figure 5D). Fasciclin 3 (Fas3) is a component of the lateral membrane strongly marking the apical position of the lateral membrane underneath the Crb-domain (Figure 5E). Fas3 amounts gradually decrease towards the basal end of the lateral membrane. In *hau* and *gho* mutant embryos the Fas3 localisation is not concentrated at the apico-lateral domain (Figure 5F,G). In conclusion, cell polarity is impaired only slightly during cuticle differentiation, and worsens until the end of embryogenesis.

Both *hau* and *gho* mutations cause defects in membrane shape. To learn to what extent membrane trafficking is abrogated by these mutations, we investigated the behaviour of small GTPases such as Rab5 and Rab11 in stage 16 and 17 *hau* and *gho* mutant embryos. Rab11 is involved in endocytic membrane recycling, whereas Rab5 regulates the fusion of endocytic vesicles with early endosomes [31], [32]. Both proteins are localised to the cytoplasm of wild-type epidermal cells with a slight accumulation to the apical portion of the cell (Figure 5A,C). In *hau* and *gho* mutant embryos the distribution of Rab5 and Rab11 is normal (Figure 5B,D).

### The tracheae of hau and gho mutant larvae

**Figure 6:**
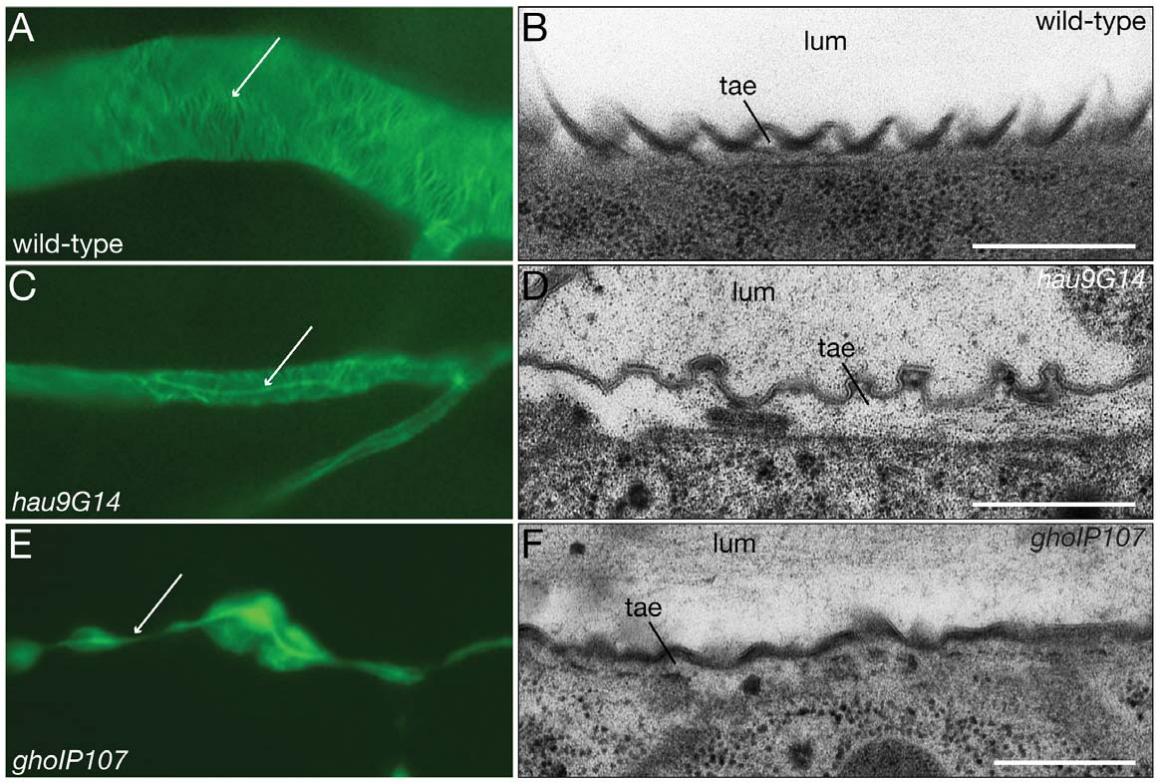
Hau and Gho are needed for the formation of the tracheal cuticle. In the wild-type tracheal cuticle of the dorsal trunk and the primary branches of late stage 17 embryos, chitin is organised in a spiral running perpendicular to the length of the tube (A). Remnants of the luminal chitin are visible (arrow). These chitin cables constitute the taenidial folds (tae), which are bulges of the larval cuticle (B). At the larval stage, the lumen (lum) of the tracheal tubes does not contain any solid material. In *hau* late stage 17 mutant embryos, the chitin cables of the dorsal trunk and the primary branches are properly formed (C). The tracheal lumen, however, is much narrower compared to the wild-type lumen. The *hau* larval tracheal cuticle dilates and the taenidial folds are sloppy (D). The lumen of the *hau* mutant larval tracheae is not completely cleared. In *gho* stage 17 mutant embryos, chitin cables are largely disorganised and often absent (E). The tracheal tubes have an irregular diameter. The *gho* mutant larval tracheae have shallow taenidiae and their lumen fails to be cleared (F). (B,D,F) Electronmicrographs. Scale bars are 500nm. (A,C,E) Fluorescence microscopy.

The tracheal cuticle covers the apical (luminal) surface of the tracheal epithelium, and forms a specialized spiral-like structure called the taenidiae. The taenidiae are chitin-containing cuticular folds that are thought to support an open lumen while allowing flexibility along the tubular axis (Figure 6A,B). Detection of chitin by CBP highlights remnants of the taenidiae in *hau* mutant embryos, whereas *gho* mutant embryos seem not to form these structures (Figure 6C and E). Ultrastructural analysis of the trachea confirms the presence of taenidiae in *hau* mutant larvae (Figure 6D), however, their size is variable and their spacing is irregular. In *gho* mutant larvae traces of shallow taenidia can be distinguished at the ultrastructural level (Figure 6F).

**Figure 7:**
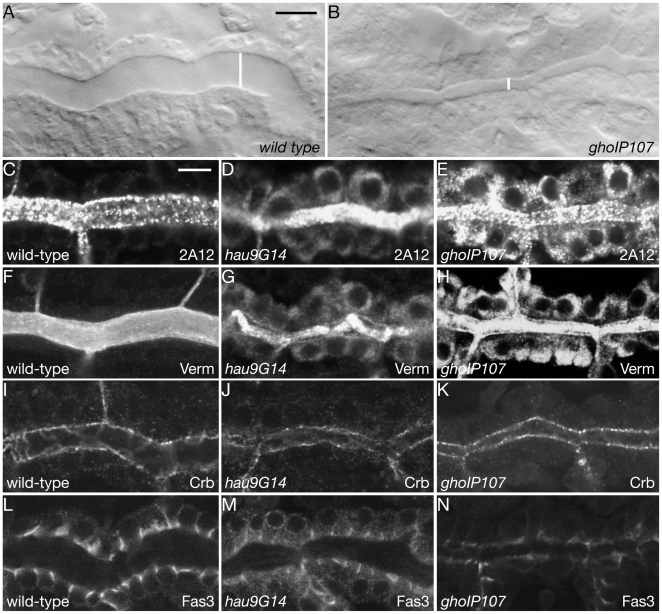
Apical secretion in tracheal cells utilizes Hau and Gho function. At early stage 17, the wild-type dorsal trunk has attained its final diameter of around 12 microns (white line, A). The early stage 17 dorsal trunks of *hau* and *gho* mutant embryos are narrower (B). During tracheal tube diameter expansion, the luminal marker 2A12 is secreted into the lumen of the tracheae (C). Most of the 2A12 signal remains within the tracheal cells of *hau* and *gho* mutant embryos during tube diameter expansion (D,E). The luminal chitin deacteylase Verm is involved in modifying the tracheal luminal chitin cable that participates in lumen diameter regulation (F). In *hau* and *gho* mutant tracheae large amounts of Verm fail to be secreted (G,H). Crb marks the apical plasma membrane in wild-type tracheal cells (I). In the tracheal cells of *hau* and *gho* mutant stage 16 embryos the localisation of Crb is unchanged (J,K). The membrane protein Fas3 lines the lateral membrane of wild-type stage 16 tracheal cells (L). In *hau* mutant embryos, some Fas3 signal is cytoplasmic (M). In *gho* mutant embryos, Fas3 localisation is as in the wild-type tracheal cells, the signal levels, however, seem to be reduced (N). Images from Confocal microscopy.

Detection of cuticular chitin also reveals that the main tracheal branches, the dorsal trunks, of *hau* and *gho* mutant larvae have a much smaller lumen diameter than those of the wild-type (Figure 6A, C and E). Such narrow lumens are also obvious in living animals (Figure 7A,B). Since lumen diameter growth is found to depend on an intact secretory pathway, and substantial apical secretion is noted in tracheal tubes during stage 15 [19], we investigated whether the narrower dorsal trunks in *hau* and *gho* mutant embryos correlate with defects in secretion. The tracheal lumen-specific antibody 2A12, recognizes a secreted product that fills the tracheal lumen from stage 14. The 2A12 signal is detected in the lumen of *hau* and *gho* mutant embryos at late stage 15, but in the mutants, there is also a strong cytoplasmic signal in the perinuclear area (Figure 7C–E). Another luminal protein, Vermiform (Verm), is a chitin-modifying enzyme detected in the dorsal trunk lumen from stage 13 [33], [34]. In addition to its detection in the lumen, the Verm signal also accumulates within the mutant tracheal cells similar to the 2A12-signal (Figure 7C–H). The secretory pathway is also responsible for the correct localisation of membrane-inserted factors. To consider this aspect, we studied the membrane factors Fas3 and Crb in stage 16 embryos (Figure 7I–N). Localisation of the apical membrane marker Crb appears normal in tracheal cells of both mutants (Figure 7I–K), but the membrane staining for Fas3 is reduced in the mutants. In wild type tracheal cells, Fas3 accumulates within the apical third of the lateral membrane (Figure 7L), whereas in *hau* mutants, this apical Fas3-staining is reduced and Fas3 is also detected in the cytoplasm (Figure 7M). In *gho* mutants Fas3 levels appear even more reduced, and Fas3 is only weakly detected at the apical domain of the lateral membrane (Figure 7N).

In summary, Hau and Gho function is important for the secretion of some tracheal luminal factors and for the stable localisation of certain membrane-inserted proteins.

### ER morphology is aberrant in the epidermal and tracheal cells of hau and gho mutant embryos

**Figure 8:**
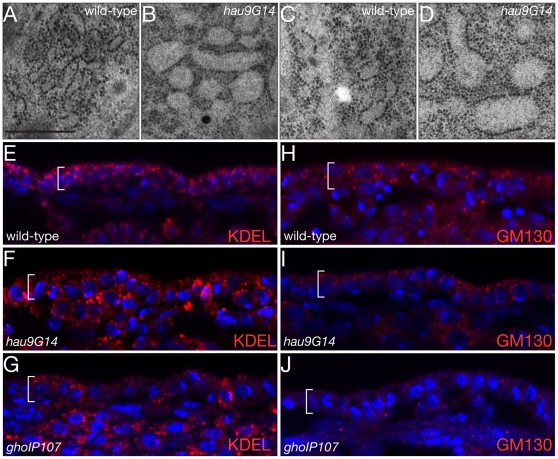
ER morphology and Golgi identity require Hau and Gho function. The wild-type embryonic stage 17 ER in the epidermal cells is tubular (A). The ER of epidermal cells in *hau* mutant stage 17 embryos has, by contrast, a bloated appearance (B). Compared to the wild-type tubular ER at stage 15 (C), the dilated ER phenotype is apparent already before massive cuticle formation (D). ER residual proteins are detected by the antibody directed against the KDEL sequence. In the wild-type stage 16 embryo the KDEL antibody recognises dots in the cytoplasm (E). In the epidermis of *hau* and *gho* mutant embryos, the KDEL signal appears to be normal (F,G). The Golgi apparatus in the wild-type stage 16 epidermis is recognised by the antibody against the Golgi-specific protein GM130 and appears as dots of different sizes (H). In the *hau* mutant stage 16 epidermis the GM130 signal is weaker (I). The GM130 is barely detected in *gho* mutant stage 16 epidermal cells (J). (A–D) Electronmicroghraphs. Scale bar in (A) is 500nm and applies also to (B–D). (E–J) Images from Confocal microscopy.

In electron micrographs, we noticed that the ER of *hau* and *gho* mutant epidermal and tracheal cells consists of large spherical compartments instead of tubules as observed in respective wild-type cells (Figure 2, 3, 6 and 7). Figures 8A and B show a magnification of the ER compartments in the epidermis of wild-type and *hau* mutant larvae, respectively, and a magnification of the ER in *hau* and *gho* mutant tracheal cells is shown in Figure S1. The perinuclear ER of these cells is affected as well (Figure S2). The epidermal and tracheal rounded ER phenotype caused by mutations in *hau* and *gho* can be traced back to stage 15, when cuticle differentiation is initiated in the epidermis (Figure 8C,D). The tracheal ER of these embryos appears to be less affected than in the epidermis (data not shown).

**Figure 9:**
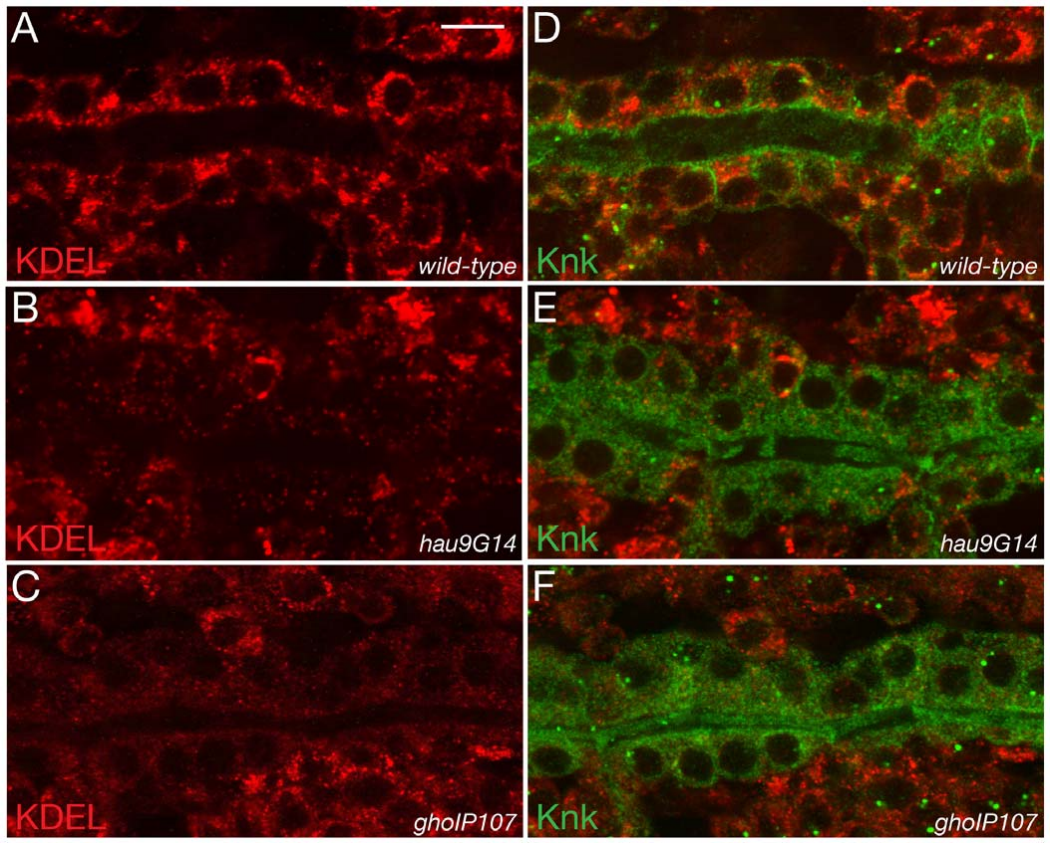
Tracheal ER identity and secretion depend on Hau and Gho function. In wild-type tracheal cells at late stage 16, the KDEL signal (red) is distributed within the cytoplasm (A,B). The membrane-associated protein Knk (green) localises to the apical plasma membrane in these cells (B). The green signal in the tracheal lumen is unspecific background. The KDEL signal in *hau* and *gho* mutant tracheal cells is strongly reduced (C–F). In both mutant tracheal cells, Knk fails to localise to the apical plasma membrane and accumulated within the cytoplasm (D,F). (A–F) Images from Confocal microscopy.

To study whether the aberrant morphology of the *hau* and *gho* mutant ER reflects a loss of ER function and identity, we performed immunohistochemical experiments to detect ER resident proteins using an antibody against the KDEL signature (Figure 8E–G). In wild-type epidermal cells of stage 16 embryos KDEL is detected as dots within the cell. KDEL detection is normal in epidermal cells of *hau* and *gho* mutant stage 16 embryos. To test whether a possible abrogated ER function may have an influence on Golgi function, we used the antibody against the Golgi organising protein GM130 to visualise the Golgi apparatus in wild-type, *hau* and *gho* mutant stage 16 embryos (Figure 8H–J). GM130 is distributed in wild-type epidermal cells as large and small dots. The GM130 signal is greatly reduced in *hau* and *gho* mutant epidermal cells. In tracheal cells of stage 16 embryos, the impact of *hau* and *gho* mutations on ER integrity is more dramatic (Figure 9). Compared to the situation in wild-type, the KDEL signal is reduced in both *gho* and *hau* mutant tracheal cells (Figure 9A–F). These experiments demonstrate that the organisation and identity of the ER and the Golgi apparatus are compromised in *hau* and *gho* embryos.

**Figure 10:**
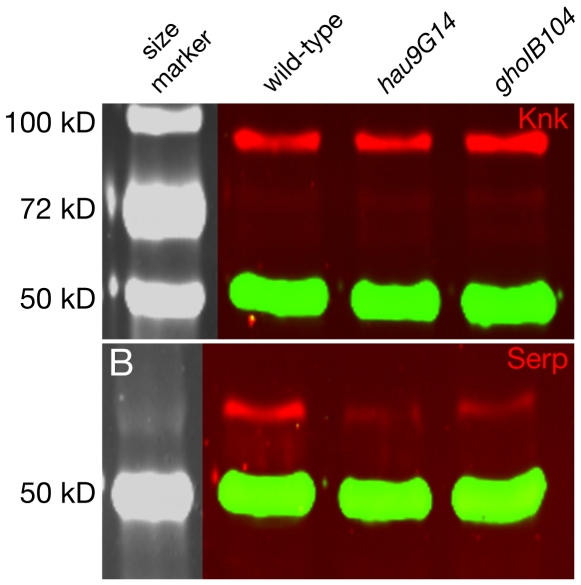
ER function is not severely affected in *hau* and *gho* mutant embryos. The migration behaviour of the membrane-associated cuticle factor Knk (red, A) and the extracellular Serp (red, B) is normal in *hau* and *gho* mutant larvae in western blot experiments. The amount of Serp protein is reduced in the mutant protein extracts. By contrast, the amounts of Knk are comparable in mutant and wild-type protein extracts. Tubulin (green) was detected to control the amount of protein blotted. The tubulin signal allows comparing the signal intensities in wild-type and mutant protein extracts.

To test whether the disrupted organisation of the ER and the Golgi apparatus in *hau* and *gho* embryos may affect correct modification of proteins running through the secretory pathway, we performed Western blot experiments using antibodies against the membrane-bound Knickkopf (Knk) protein, or the secreted Serp protein (Figure 10). The size of Knk, that we have previously shown to be glycosylated at three sites [13], [15], is unchanged in *hau* and *gho* mutant embryos (10A). The migration of the extracellular Serp protein with three putative N-glycosylation sites (Asp^246^, Asp^276^, Asp^298^) is also normal in these embryos (10B). Hence, ER and Golgi organisation but not their function as compartments of N-glycosylation are compromised by mutations in *hau* or *gho*.

### hau and gho encode factors of the COPII complex

**Figure 11:**
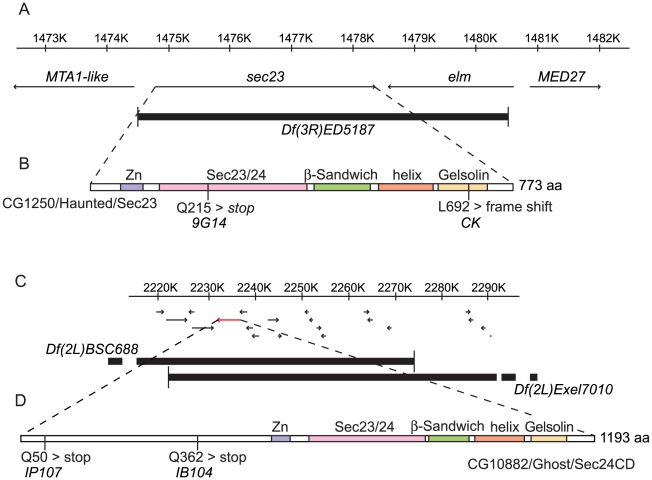
Molecular identification of *hau*. The *hau* mutations were mapped to the deficiency Df(3R)ED5187 that uncovers two genes: *sec23* and *elm* (A). Sec23 is a component of the COPII complex. The *elm* gene plays a role in memory formation and ethanol sensitivity, and mutations in this gene are not lethal [66]. A nonsense mutation was identified in the *sec23*-coding region of the *9G14* allele, and a frame shift mutation was identified in the same coding region of the *CK* allele (B). Sec23 is characterised by five motifs. From the N-terminus to the C-terminus, these are the Zn binding domain, the Sec23/24 domain, which belongs to the von Willebrand factor type A (vWFA) domain family, a β-sandwich domain, a helical domain and finally a Gelsolin domain. Molecular identification of *gho*. The *gho* mutations were mapped to the interval framed by the break points of the deficiencies Df(2L)BSC688 and Df(2L)Excel7010 (C). Among the 16 genes in this region, one codes for a Sec24-like protein, CG10882 (D). In the coding region of this gene, we identified one early nonsense mutation in each allele, *IB104* and *IP107*.

To understand the molecular roles of *hau* and *gho* in cuticle differentiation, we identified the genomic location and the sequence of both genes. Mutations in *hau* had previously been mapped to the right arm of chromosome 3 (cytological location 85D) [35], and mutations in *gho* had been localised to the right arm of chromosome 2 (recombination map position 68) [24]. By deficiency mapping, we localised *hau* to the cytological interval between 83B7 and 83B8 uncovered by the deficiency Df(3R)ED5187 on the right arm of chromosome 3 (Figure 11A), and *gho* to the cytological interval between 22D4 and 22D6 defined by the overlapping region of the deficiencies Df(2L)Excel7010 and Df(2L)BSC688 on the left arm of chromosome 2 (Figure 11C). Thus, the mapping data in [24] and [35] are inaccurate. The *hau*-containing interval harbours two genes, one of which is CG1250 that encodes the only *Drosophila* Sec23 ortholog, that, as a COPII component, is involved in vesicle budding from the ER [36]. We sequenced the *sec23* genomic DNA of embryos homozygous mutant for *hau* and detected a point mutation in each of our alleles (Figure 11B). A transition of the C^643^ to T resulting in a nonsense mutation changing Gln^215^ to *amber* (TAG) was detected in the *hau^9G14^* allele. This mutation disrupts the Sec23/24 or von Willebrand factor type A (vWFA)-like domain in the first half of the protein and deletes all consecutive domains. The P-element induced allele [35] has a deletion of G^2076^ of the coding sequence causing a frame shift that changes the peptide sequence after Lys^692^. This mutation leads to the elimination of Arg^722^, which is essential for the interaction of Sec23 with Sar1, the GTPase that triggers vesicle budding from the ER [37], [38]. The *Drosophila* Sec23 protein is over its entire length 73% identical and 85% similar to the human Sec23 protein (isoform A).

A gene coding for a *Drosophila* Sec24-like protein, which interacts with Sec23 [38], is located in the *gho*-containing interval (*CG10882*) enclosing 16 genes (Figure 11C). Due to the phenotypic resemblance of *hau* and *gho* mutant embryos, we considered this gene as a candidate to be mutated in *gho* mutant embryos. We sequenced the respective genomic DNA and identified base-pair changes in both *gho* EMS alleles (Figure 11D). The 50^th^ codon in *IP107* (CAG) is mutated to an *amber* nonsense codon and the respective protein stops after Gln^49^. The mutation therefore results in a short protein that lacks the functional domain of the Sec24-like protein. Because of an *amber* nonsense mutation of codon 362 (CAG), the IB104 protein ends at residue Pro^361^. This protein lacks the complete domain set common to Sec23/Sec24 proteins, including the Sec23/24 (vWFA-like) domain, and only consists of a region which is specific to this Sec24 paralog and which is predicted to be unstructured. Besides CG10882 the *Drosophila* genome harbours another gene (CG1472) encoding a Sec24-like protein, which is actually annotated as the *Drosophila* Sec24 ortholog. The two *Drosophila* Sec24 proteins correspond to four human orthologs. The *Drosophila* Sec24 protein CG10882 is rather similar to the human orthologs Sec24C and D than to Sec24A and B, which share high similarity to CG1472 (Figure S2). Following these findings, we consider CG10882 as the *Drosophila* Sec24CD and CG1472 as the *Drosophila* Sec24AB paralogs.

The mutations in the *Drosophila sec23* (*hau*) and *sec24cd* (*gho*) genes most probably disrupt protein function resulting in embryo lethality. We therefore conclude that *hau* and *gho* code for Sec23 and one of the *Drosophila* Sec24 paralogs, respectively, both being components of the COPII complex. In a recent article, Förster and colleagues reported on the role of CG10882, named Stenosis (Sten) during tracheal development [39]. Consistently, mutations in *sten* fail to complement mutations in *gho* (data not shown). Since mutations in *gho* were identified earlier [24], we continue denoting *CG10882 gho*. Despite the evident importance of both factors for cell viability, animals mutant for either factor die rather late during embryogenesis (Figure 1); this observation can be explained by the presence of maternally provided function supporting development until the end of embryogenesis [39], [40].

### Mutations in genes encoding COPII components cause similar phenotypes

**Figure 12:**
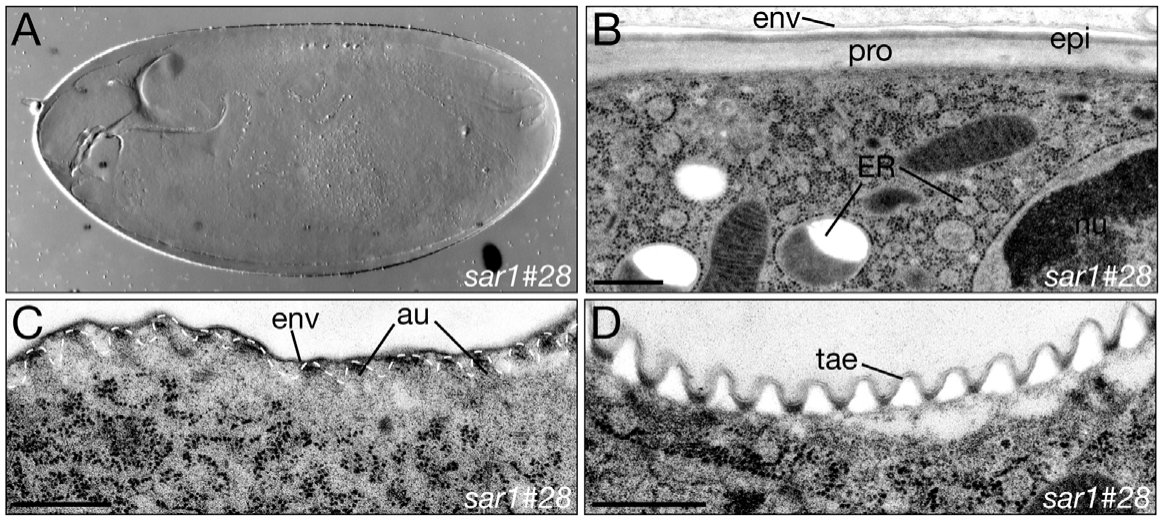
Deletion of *sar1* causes a weak secretion phenotype. Larvae having a deletion of the *sar1* locus have a thin and pale cuticle (A). The three histologically distinct layers, envelope (env), epicuticle (epi) and procuticle (pro) are established (B). The ER in the epidermal cells of *sar1* mutant larvae is bloated. The apical undulae (au, dotted line) of stage 16 *sar1* mutant embryos are formed (C). Likewise, the taenidial folds (tae) are correctly established in *sar1* deficient larvae (D). (A–D) Electron micrographs. Scale bars in (B,C,D) are 500nm.

Sec23 and Sec24 form a complex with Sar1, a GTPase that triggers membrane budding from the ER [41], [42]. Mutations in the *Drosophila sar1* have been reported to abrogate secretion in tracheal and epidermal cells [19], [43]. Macroscopically, larvae with a deletion of the first four exons of the *sar1* gene [44] display a weaker phenotype than larvae mutant for *hau* and *gho* (Figs. 1 and 12). For a detailed comparison of *sar1*, *hau* and *gho* phenotypes, we examined the ultrastructure of the epidermis of *sar1* mutant larvae that are characterised by a pale cuticle (Figure 12A). A stratified cuticle is formed in *sar1* mutant larvae, which is however thinner than the wild-type cuticle (Figure 12B). As in *hau* and *gho* mutant larvae and consistent with recently published data [19], the ER of the epidermal cells of *sar1* mutant larvae is spherical. Apical undulae formation is unaffected by *sar1* mutations (Figure 12C). Moreover, the taenidia of the *sar1* mutant tracheae are normal (Figure 12D). In summary, the *sar1* mutation causes a weak COPII-deficient phenotype.

## Discussion

Epithelia are composed of polarised cells and possess extracellular matrices at their apical and basal sides. In this article, we show that in the *Drosophila* embryonic epidermis and tracheal epithelium the COPII components Sec23 and Sec24 stabilise cell polarity, are needed for tracheal tube diameter growth and mediate formation of the apical and basal ECMs.

### hau and gho code for the COPII components Sec23 and Sec24CD

Hau and Gho represent the only *Drosophila* Sec23 and one of the two *Drosophila* Sec24 proteins, respectively, which are components of the COPII complex that coats vesicles transporting ER products to the Golgi apparatus [45]. Sec24 is responsible for specific cargo recognition and binding. In yeast, for instance, several Sec24-like proteins such as Iss1p and Lst1p act in parallel to ensure correct delivery of cargo proteins to their destination [46]. Sec23 is a central molecule of the COPII complex [36]. Through the interaction with Sec24, Sec23 links cargo recognition to vesicle budding [47], and through binding to dynactin it links vesicle budding to vesicle transport [48]. Sec23 and Sec24 are essential proteins in eukaryotic cells from yeast to humans [49]. In yeast, depletion of Sec23 blocks protein transport [50]. A hypomorph mutation in the human Sec23A-coding gene has been shown to cause craniofacial morphological abnormalities termed Cranio-lenticulo-sutural dysplasia (CLSD) [49], [51]. Consistently, zebrafish embryos carrying mutations in *bulldog* that codes for the Sec24D paralog, suffer skeletal dysmorphologies caused by the failure to secret ECM material [52]. In a recent article, Förster and colleagues reported on the requirement of *Drosophila* Sec24CD, which they call Stenosis (Sten), for tracheal cell shape changes and tube expansion in a cell-autonomous manner [39].

Besides Sec23 and Sec24, the inner coat of COPII vesicles also harbours the small GTPase Sar1 that initiates vesicle formation. Mutations in *sar1* cause a similar albeit weaker, phenotype compared to those caused by *hau* and *gho* mutations. Indeed, regarding the cuticle, we observe a phenotypic series from strong defects in *gho* mutant larvae, over moderate defects in *hau* mutant larvae to weak defects seen in *sar1* mutant larvae. The differences in consequences of presumably null mutations in all three genes derive probably from the stability and endurance of the respective maternally provided gene product. In this view, the virtual absence of the body cuticle in *gho* mutant larvae indicates that the function of the Sec24CD protein is almost completely eliminated in late embryonic stages and late secretion is utterly interrupted. One may conclude that the two *Drosophila* Sec24 paralogs do not have redundant functions in the epidermis and that the second Sec24-like protein, i.e. Sec24AB, does not play any role in epidermal cuticle formation.

### Hau and Gho are needed for ER function and integrity

Blocking of COPII vesicle transport by mutations in *sec23*, *sec24* and *sar1* causes a stop of protein trafficking to the extracellular space or to the plasma membrane, and concomitantly, the ER loses its tubular shape and becomes spherical. This subcellular phenotype is also observed in human *SEC23^−/−^* fibroblasts and in HeLa cells deficient for Sec13, which is part of the COPII coat [36], [53].

The loss of ER morphology may be due to the excess accumulation of not transported proteins that may physically destroy the molecular corset of the ER. This would imply that a mechanism controlling the amount of proteins in the ER does not exist. In other words, ER exit sites and ER loading complexes do not seem to communicate.

Overloading of the ER may downgrade ER physiology, and thereby interfere with the activity of ER specific enzymes. One may speculate that this in turn may induce ER stress that leads to the degradation of ER proteins [54]. Our data on this issue are conflicting. On the one hand, the amounts of Serp and ER residual proteins detected by the KDEL antibody seem to diminish in *hau* and *gho* mutant embryos, which on the other hand have normal amounts of Knk although it does not reach the plasma membrane. Hence, this type of ER dysfunction seems to elicit degradation or exhaustion of selective target proteins, at the same time sparing others.

### COPII function is necessary and sufficient to drive cuticle production

The defects provoked by reduction or elimination of Sec24CD activity illustrate the COPII-deficient phenotype in the tracheae and the epidermis. Hence, structuring of all three layers of the cuticle depends on ER-to-Golgi transport via COPII coated vesicles. The Sec24CD dependent COPII vesicles, thus, carry cuticle components as diverse as for example the chitin synthase producing the bulk of the procuticle, factors of the extracellular melanisation pathway, and the yet unknown lipid-handling enzymes of the envelope and epicuticle. Of course, this does not exclude that COPII-independent routes are employed to modify layer architecture. For instance, transcytosis of phenoloxidases from the haemolymph to the cuticle [55] does not utilise the canonical secretory pathway and therefore is probably not directly affected by COPII-deficiency. It may well be, however, that disruption or weakening of cell polarity induced by *gho* mutations indirectly interferes also with alternative secretory routes [56], [57].

How may a single secretory route accommodate asymmetric distribution of factors within the plasma membrane and within the extracellular space? From the conclusion that most, if not all, cuticle factors are recruited by the ER-COPII-Golgi path for delivery to the apical plasma membrane or extracellular space, it follows that cargo divergence has somehow to occur within the route itself. Indicated by the finding that the chitin synthase complex localises to the apical plasma membrane independently from the t-SNARE Syntaxin1A [18], one potential site of divergence is the apical plasma membrane itself, where different t-SNAREs may occupy different domains. Sorting is also a central task of the Golgi apparatus, where vesicles with distinct cargos are generated [58]. At the COPII level, cargo divergence possibly engages p24 proteins, which in yeast have been reported to recognise specific cargos [59]. A major endeavour in the near future will be to elucidate the sorting mechanisms in the secretory pathway that drive cuticle differentiation i.e. aECM formation.

### Basement membrane formation requires COPII function

Concomitant with cuticle deposition and organisation the basement membrane is produced during *Drosophila* embryogenesis [29]. Hence, in parallel to the coordination of apical secretion, epithelial cells have to control basal secretion as well. In *hau* and *gho* mutant embryos, the basement membrane is practically missing, suggesting that a distinct population of COPII vesicles is charged with basal ECM formation. The handling of these vesicles is, however, not sufficient to form a functional basement membrane, as in addition to the epithelial contribution, some components of the basal ECM such as collagen IV are supplied by macrophages [60]. Nevertheless, epithelial cells appear to retain sovereignty over this process. For example, Scarface (Scarf), a serine-protease-like protein and Crag, a factor associated with the secretory pathway, have been proposed to regulate the localisation of basement membrane components and act within the epithelium itself [61], [62]. The loss of basal factors in *hau* and *gho* mutant embryos, could, in agreement with the finding that the basal ECM is essential for epithelial cell polarity [63], [64], at least partially explain the loss of epithelial character in these animals.

### Plasma membrane topology in the epidermal cell depends on Hau and Gho function

The *Drosophila* COPII components Sec23 and Sec24CD are not only mediating cuticle deposition and basal ECM assembly, they are also important for shaping the apical plasma membrane. In contrast to the longitudinal corrugations, the apical undulae, that are presumably essential for organising the cuticle in the wild-type embryo [29], the apical plasma membrane of *hau* and *gho* mutant embryos fails to corrugate. The epidermal apical membrane is flat also in embryos mutant for the apical plasma membrane tSNARE Syx1A [18]. Hence, the topology of the apical plasma membrane requires the canonical secretory pathway running from the ER via COPII vesicles to the Golgi apparatus and from the Golgi apparatus to the apical plasma membrane where Syx1A is controlling incorporation of membrane material.

Likewise, during cuticle differentiation, Sec23 and Sec24CD serve for the maturation of the lateral plasma membrane from a straight to a meandering structure where different protein complexes assemble along its apico-basal domains, among others enforcing cell-cell contacts [65]. The lateral plasma membrane of *hau* and *gho* mutant larvae remains straight and, as suggested by their less electron-dense appearance in electronmicrographs, the SJs are depleted from proteins arguing that cell polarity is perturbed. Weakened cell polarity supposedly corrupts the arrangement of the cytoskeleton, in turn affecting polarised secretion. At the end of such a vicious circle single naked epidermal cells round up and leave the epithelium as observed especially in *gho* mutant larvae. This finding is in agreement with the recently formulated notion that cell polarity is not a stable state of the cell but requires continuous recycling of Crb via a Rab11-dependent mechanism [9]. Taken together, the secretion pathway plays a central role during differentiation of the *Drosophila* larval epidermis coordinating its primary task of cargo transport and membrane trafficking with maintenance of cell polarity.

## Supporting Information

Figure S1 Hau and Gho function contributes to the morphology of the ER of the tracheal cells. The ER of wild-type larval tracheal cells is tubular (A). By contrast, the ER of hau and gho larval tracheal cells is dilated (B,C). The ER enveloping the nucleus of the wild-type larval epidermal cell is tightly following the shape of the nucleus itself (D). In hau larvae, the perinuclear ER forms cysts (E). In gho larvae, this phenotype is similar (not shown). (A–E) Electron micrographs. Scale bar in (A,D,E) is 500 nm. The scale bar in (A) applies also to (B) and (C).
(2.47 MB TIF)

Figure S2 Schematic sequence comparison between Drosophila and human Sec24 paralogs. Sequences were compared using pairwise BLAST (http://blast.ncbi.nlm.nih.gov/Blast.cgi). The pairwise identity scores from BLAST are given in the colored bars; the size of the bars illustrates the alignable sequence regions. Alignable stretches of less than 30 residues were ignored. Clearly, Drosophlia CG10882 (Gho) has higher local and global similarity to human Sec24C, Cb (a splice variant of C) and D, while Drosophila CG1472 displays higher similarity to the human Sec24A and B paralogs.
(0.46 MB EPS)
