# Supplementary figures and images for: Trafficking through COPII Stabilises Cell Polarity and Drives Secretion during Drosophila Epidermal Differentiation
Source: PLoS One. 2010 May 24;5(5):e10802. doi: 10.1371/journal.pone.0010802 (PMC2875407; doi:10.1371/journal.pone.0010802)

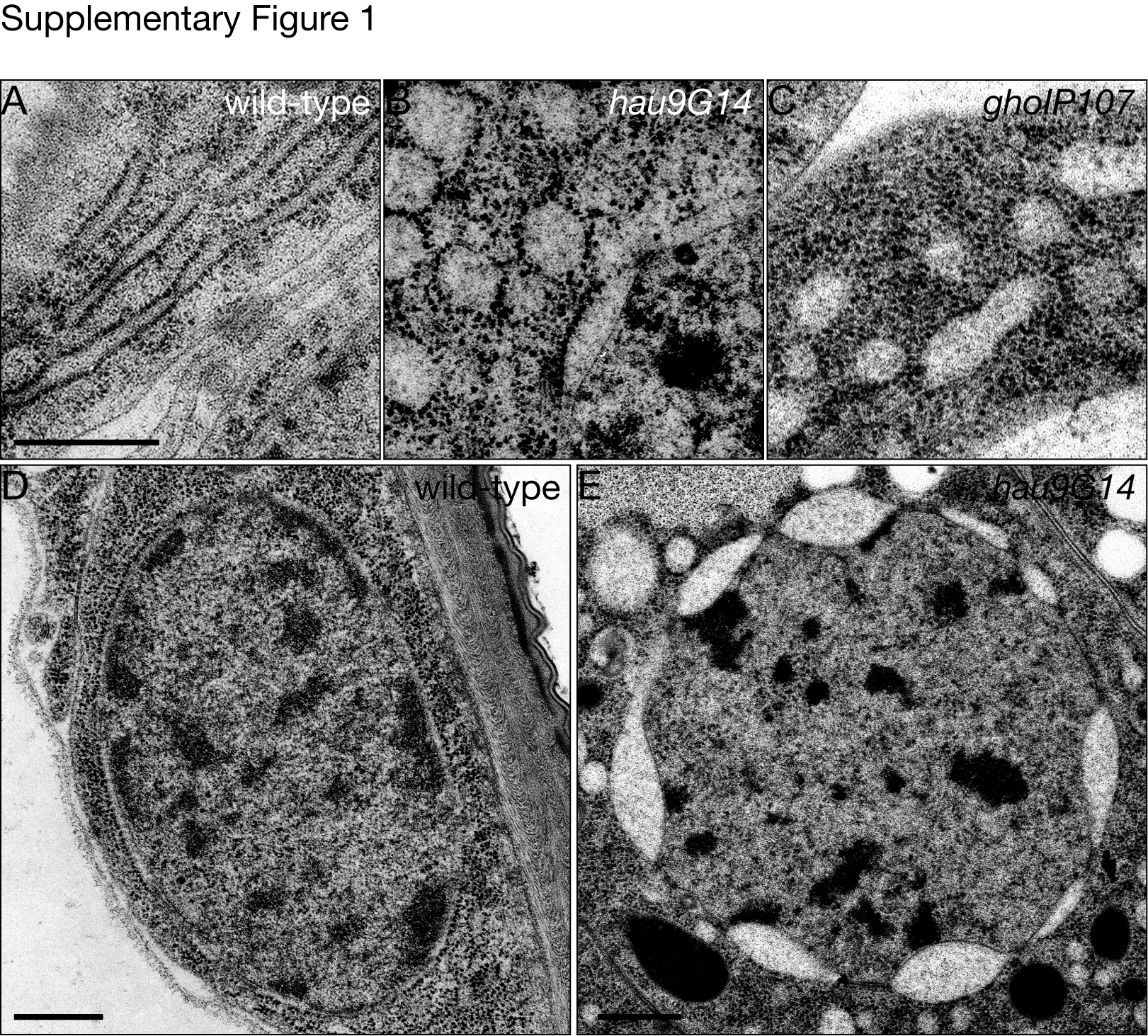

Supplement: Figure S1 — Hau and Gho function contributes to the morphology of the ER of the tracheal cells. The ER of wild-type larval tracheal cells is tubular (A). By contrast, the ER of hau and gho larval tracheal cells is dilated (B,C). The ER enveloping the nucleus of the wild-type larval epidermal cell is tightly following the shape of the nucleus itself (D). In hau larvae, the perinuclear ER forms cysts (E). In gho larvae, this phenotype is similar (not shown). (A–E) Electron micrographs. Scale bar in (A,D,E) is 500 nm. The scale bar in (A) applies also to (B) and (C). (2.47 MB TIF) [file pone.0010802.s001.tif]
